# Serotype distribution and antimicrobial susceptibility of invasive *Streptococcus pneumoniae* isolates among adult and elderly population before and after introduction of pneumococcal conjugate vaccine in Casablanca, Morocco

**DOI:** 10.1186/s12879-023-07981-y

**Published:** 2023-01-13

**Authors:** Néhémie Nzoyikorera, Idrissa Diawara, Mostafa Katfy, Khalid Katfy, Fakhreddine Maaloum, Joseph Nyandwi, Houria Belabbes, Naima Elmdaghri, Khalid Zerouali

**Affiliations:** 1 Department of Microbiology, Faculty of Medicine and Pharmacy of Casablanca, Hassan II University of Casablanca, Casablanca, Morocco; 2 Higher Institute of Biosciences and Biotechnology, Mohammed VI University of Health Sciences (UM6SS), Casablanca, Morocco; 3 Laboratory of Microbial Biotechnology and Infectiology Research, Mohammed VI Center for Research & Innovation, Rabat, Mohammed VI University of Health Sciences (UM6SS), Casablanca, Morocco; 4 National Reference Laboratory, National Institute of Public Health, Bujumbura, Burundi; 5 Bacteriology-Virology and Hospital Hygiene Laboratory, Ibn Rochd University Hospital Centre of Casablanca, Casablanca, Morocco; 6 Département de Médecine, Faculté de Médecine, Université du Burundi, Bujumbura, Burundi; 7 Ministère de la Santé Publique et de la Lutte contre le Sida, Institut National de Santé Publique de Bujumbura, Bujumbura, Burundi

**Keywords:** *Streptococcus pneumoniae*, Serotype, Antibiotic resistance, Invasive pneumococcal diseases, Adult population, Herd effect, Pneumococcal conjugate vaccine

## Abstract

**Background:**

*Streptococcus pneumoniae (S. pneumoniae)*, remains a major cause of mortality and morbidity worldwide. The objective of this study was to determine the trends of invasive pneumococcal diseases (IPD) in adult and elderly population in Casablanca (Morocco) before and after introduction of pneumococcal conjugate vaccine (PCV) by determining the distribution of pneumococcal serotypes and antibiotic resistance profile of isolated strains.

**Method:**

The proposed study is a retrospective laboratory-based surveillance of IPD in hospitalized adult (15–59 years old) and elderly (≥ 60 years old) patients in Ibn Rochd University Hospital Centre from 2007 to 2019 (13 years). All the 250 non-duplicate clinical invasive isolates from adult and elderly patients, confirmed as *S. pneumoniae* according to the laboratory standard identification procedures, are included in this study.

**Results:**

A significant decrease of the overall incidence in IPD was observed only in adults from 0.71 to 0.54/100000 populations (*P*  =  0.02) and to 0.47/100000 populations (*P*  =  0.0137) in the early and mature post-vaccine period respectively compared to the pre-vaccine period. Our results also showed a significant reduction in the overall prevalence of vaccine serotypes from 28.17 to 6.90% (*P*  =  0.0021) for the PCV-10 serotypes, and from 46.48 to 25.86% (*P*  =  0.0164) for the PCV-13 serotypes only in the mature post-vaccine period (2015–2019). In parallel, the rate of non-vaccine serotypes did not significantly change in the early post-vaccine period (2011–2014) while it increased considerably from 54 to 74.14% (*P*  =  0.0189) during the mature post-vaccine period. The rate of penicillin non-susceptible pneumococcal isolates decreased significantly from 23.94 to 8.77% (*P*  =  0.02) in adult patients, and the rate of cotrimoxazole non-susceptible pneumococcal isolates significantly decreased from 29.58 to 8.77% in the early post-vaccine period (*P*  =  0.003) and to 7.24% in the mature post-vaccine period (*P*  =  0.0007).

**Conclusion:**

Although childhood vaccination has considerably reduced the incidence of IPD in adult population through the herd effect, IPD remain a real public health problem due to the alarming increase in non-vaccine serotypes (NVS) and the lack of herd effect among elderly population. The rate of antibiotic resistance was relatively low. Nevertheless, resistance constitutes a serious problem to the therapeutic arsenal due to the known capacity for genetic dissemination in the pneumococcus.

## Background

*Streptococcus pneumoniae (S. pneumoniae)*, also known as pneumococcus, is an encapsulated Gram-positive bacterium belonging to the genus *Streptococcus* and the family *Streptococcaceae*. A commensal bacterium of the human nasopharynx, the pneumococcus can pass from the status of asymptomatic carriage to that of a major pathogen and cause of non-invasive diseases such as sinusitis, otitis media, conjunctivitis and invasive infections such as meningitis, bacteraemia, bacteraemic pneumonia and pleural empyema. It is one of the main pathogens responsible for morbidity and mortality worldwide [1]. Globally, 294,000 of 5.83 million deaths among < 5 year old children were related to pneumococcal infections in 2015 according to the World Health Organization (WHO) [2].

The management of invasive infections mainly includes treatment with antibiotics and prevention through the use of vaccines which is the best option for preventing invasive pneumococcal diseases (IPD). In several countries, pneumococcal conjugated vaccines (PCV) have been recommended in childhood immunization programs for more than a decade. In Morocco, the National Immunization Program (NIP) introduced the PCV-13 in October 2010 in 2 + 1 schedule at 2, 4 and 12 months of age, and switched to PCV-10 in July 2012. They have significantly reduced the global burden of IPD in children as reported in the review of Izurieta [3]. Nowadays, 100 different serotypes have been identified and characterized [4]. However, the serotype prevalence and distribution vary greatly depending on geographic region, patient age, vaccination coverage, and time period of surveillance, which implies the need for continuous surveillance of pneumococcal infections.

Beta-lactam antibiotics, especially the third-generation cephalosporins (C3G), are used to treat the pneumococcal infections. Other antibiotic families (macrolides, lincosamides, fluroquinolones) are needed for beta-lactam resistant pneumococcal isolates or for patients who do not tolerate beta-lactams. Globally, the increase of penicillin-non-susceptible pneumococci (PNSP) have been documented in various regions [5, 6]. The emergence of the antimicrobial drug resistant pneumococcal isolates constitutes a serious therapeutic problem. Since the resistance patterns of *S. pneumoniae* vary according to geographic location, time, age, site of infection, and serotypes in circulation, the surveillance must be implemented and the guidelines must be derived from the local epidemiology [6, 7]. In Casablanca, pneumococcal laboratory-based surveillance has started since 1994 in Ibn Rochd University Hospital Centre (IR-UHC) of Casablanca. The published results on serotype distribution and antimicrobial susceptibility of *S. pneumoniae* strains isolated until 2014 targeted mostly the children < 5 years [7–11]. They showed an overall decrease of vaccine serotypes (VS) among children < 2 years as well as a slight increase in non-vaccine serotypes (NVS) and significant decrease of the PNSP strains [9]. The main objective of this study was to determine the trends of IPD in adult and elderly population in Casablanca, Morocco before and after introduction of PCV in the national immunization program. The secondary objectives are to determine the distribution of pneumococcal serotypes responsible for IPD and the antimicrobial susceptibility pattern of isolated strains and their antibiotic resistance profile.

## Methods

### Study population

The proposed study is a laboratory-based surveillance of IPD in hospitalized adult (15–59 years old) and elderly (≥ 60 years old) patients in IR-UHC. This centre is the only and well-known public tertiary hospital covering the entire region of Grand Casablanca, where almost all cases of serious diseases such as meningitis and complicated diseases in other hospitals are systematically transferred. It is composed of four training hospital formations covering all ages and all specialties. The microbiology department belongs to the laboratory of the IR-UHC, ensuring all microbiological analyzes. The Grand Casablanca with the same 4 provinces as before the territorial division of 2015 (Casablanca, Mohammedia, Nouaceur, Mediouna) is located on the Atlantic coast, in the center west of the country. This region covers an area of 1140.54 km^2^. It is the most populated region of the Kingdom of Morocco with a population of 4,648,720 in 2018, which represents 13.19% of the national population of Morocco as provided by the High Council for Planning of the National Centre of documentation in Morocco [12].

### Study design

This is a retrospective study to evaluate the trend of IPD before and after introduction of PCV in Moroccan NIP in adult and elderly patients hospitalized in IR-UHC from 2007 to 2019 (13 years). All clinical invasive isolates from adult and elderly patients that were confirmed as *S. pneumoniae* according to the laboratory standard pneumococcal identification procedures are included in this study. All duplicate *S. pneumoniae* isolates are not considered in this study. To assess the impact of PCV in these two categories of age, the study period was subdivided intopre-vaccine period (2007–2010) and post-vaccine period (2011–2019). The post-vaccine period was subdivided into early post-vaccine period (2011–2014) and mature post-vaccine period (2015–2019) based on vaccine coverage rate.

### Pneumococcal isolates

All *S. pneumoniae* isolates were identified following standard procedures of bacteriology (i.e., α-hemolysis, optochin susceptibility and bile solubility). Each pneumococcal isolate was reported on a laboratory report form including demographic and medical information on the patient. All isolates of *S. pneumoniae* from a normally sterile body site (e.g., blood, cerebrospinal fluid (CSF), or, less commonly, joint, pleural or pericardial fluid) were categorized as IPD. When an isolate was recovered from CSF and blood, the case was categorized as meningitis [9]. From 2007 to 2019, all the non-duplicate invasive *S. pneumoniae* isolates recovered from adult and elderly patients were included in this study.

### Pneumococcal serogrouping/serotyping

Serogrouping was performed by the checkerboard method with Pneumotest-latex (Staten’s Serum Institute antisera, Copenhagen, Denmark). Serotyping was performed by Quellung capsule swelling method using Statens Serum Institute antisera (Copenhague, Denmark). For some strains, serogrouping/serotyping were also done by multiplex PCR as described previously by Centers for Disease Control and Prevention (CDC) [13].

### Antimicrobial susceptibility

Antimicrobial susceptibility by disc diffusion for 5 antibiotics (erythromycin, chloramphenicol, co-trimoxazole, tetracycline and levofloxacin) and determination of the minimum inhibitory concentration (MIC) of penicillin G and ceftriaxone with E-tests (Oxoid, Basingstoke, UK) were carried out on Mueller–Hinton agar with 5% of sheep blood (BioMérieux, Marcy-l’Etoile, France) and interpreted according to the Clinical and Laboratory Standards Institute (CLSI,2012) [14]. recommendations before 2014 and the European Committee on Antimicrobial Susceptibility Testing (EUCAST) recommendations after 2014. Oxacillin (1 µg) was used for screening of PNSP isolates. Quality control was conducted using *S. pneumoniae* ATCC 49,619.

### Statistical and data analysis

Computerized data was analyzed with Epi Info version 7.1.5 and Microsoft Excel version 2019. To better assess the impact of vaccination on serotypes, different serotypes identified were subdivided into three major groups: VS group covered by PCV-10 and/or PCV-13 and NVS group not covered by PCV-13 [9]. The annual incidence rate of IPD per 100,000 populations was estimated by dividing the number of confirmed infections by the number of populations of the target group (15–59 years and ≥ 60 years) per year. The chi square test or Fisher’s exact test were performed to compare proportion between different periods [9]. Statistical significance was determined with 95% confidence interval (CI). Change in incidence rate between the pre- and post-vaccination periods was assessed by calculating absolute risks reduction and relative risks reduction (ARR and RRR) [15, 16]. Differences were considered significant if the *P* < 0.05.

## Results

### Isolate information and incidence rate of IPD

A total of 250 isolates was recovered from adult (n  =  197) and elderly patients (n  =  53) during our study period (from 2007 to 2019), among them 34.4% (n  =  86), 33.2% (n  =  83) and 32.4% (n  =  81) were recovered from the pre-vaccine period, the early and mature post-vaccine period respectively. Compared to the pre-vaccine period, a significant decrease in the overall incidence rate of IPD in the adult population was observed, ranging from 0.71 to 0.54/100,000 populations (*P*  =  0.02) with a decrease rate of 24.18% (IC 95%  =  − 55.51 to + 6.33) and from 0.71 to 0.47/100,000 populations (*P*  =  0.0137) with a reduction rate of 66% (95% CI: 46% to 93%) in the early and mature post-vaccine period, respectively (Table 1). As for the elderly patient group, the incidence rate underwent a non-significant increase from 1.28 to 2.09/100,000 populations (*P*  =  0.12) in the early post-vaccine period while it decreased to 0.69/100,000 populations (*P*  =  0.11) in the mature post-vaccine period compared to the pre-vaccine period.

**Table 1:** Sample origin and IPD incidence according to age groups in the pre-and post-vaccination periods

	Pre-vaccine period 2007–2010 no. of cases/100,000 populations^a^	Post-implementation no. of cases/100.000 populations^b^		Baseline (2007–2010) vs early post-vaccine period (2011–2014)			Baseline (2007–2010) vs mature post-vaccine period (2015–2019)		
		Early post-vaccine period 2011–2014	Mature post-vaccine period 2015–2019	Absolute risk reduction cases/100,000 populations (95% CI)	Relative Risk Reduction % (95% CI)	*p-*value	Absolute risk reduction cases/100,000 populations (95% CI)	Relative risk reduction % (95% CI)	*p-*value
*15–59 years*									
CSF	0.18	0.09	0.26	− 0.09 (− 0.20 to + 0.02)	− 47.53 (− 110.37 to + 9.88)	0.09	− 0.09 (− 0.20 to + 0.02)	0.73 (0.45 to 1.20)	0.19
Blood	0.35	0.37	0.16	+ 0.02 (− 0.15 to + 0.19)	+ 5.23 (− 42.98 to + 52.99)	0.82	+ 0.02 (− 0.15 to + 0.19)	0.86 (0.44 to 1.71)	0.65
Pleural fluid	0.07	0.04	0.03	− 0.03 (− 0.11 to 0.04)	− 46.03 (− 157.46 to + 53.22)	0.48	− 0.03 (− 0.11 to 0.04)	0.48 (0.12 to 1.77)	0.21
^c^Others	0.11	0.04	0.02	− 0.07 (− 0.16 to 0.00)	− 65.65 (− 147.48 to + 4.07)	0.09	− 0.07 (− 0.16 to 0.00)	0.18 (0.03 to 0.70)	0.0037
Total	0.71	0.54	0.47	− 0.17 (− 0.39 to + 0.04)	− 24.18 (− 55.51 to + 6.33)	0.02	− 0.17 (− 0.39 to + 0.04)	0.66 (0.46 to 0.93)	0.0137
* ≥ 60 years*									
CSF	0.77	0.56	0.23	− 0.20 (− 0.95 to + 0.50)	− 26.55 (− 124.25 to + 64.88)	0.53	− 0.20 (− 0.95 to + 0.50)	0.68 (0.12 to 3.65)	0.58
Blood	0.34	1.12	0.17	+ 0.78 (+ 0.08 to + 1.58)	+ 230.55 (+ 24.26 to + 463.05)	0.04	+ 0.78 (+ 0.08 to + 1.58)	0.22 (0.03 to 0.90)	0.0149
Pleural fluid	0.09	0.24	0.23	+ 0.16 (− 0.27 to + 0.63)	+ 183.32 (− 319.24 to + 739.21)	0.62	+ 0.16 (− 0.27 to + 0.63)	2.72 (0.26 to 134.03)	0.35
Other	0.09	0.16	0.06	+ 0.08 (− 0.34 to + 0.51)	+ 88.88 (− 397.33 to + 595.49)	1	+ 0.08 (− 0.34 to + 0.51)	0.68 (0.008 to 53.41)	0.78
Total	1.28	2.09	0.69	+ 0.81 (− 0.25 to + 1.91)	+ 63.70 (− 19.53 to 149.45)	0.12	+ 0.81 (− 0.25 to + 1.91)	0.54 (0.23 to 1.24)	0.11

Incidences were calculated as incidence  =  Number of IPD cases × 100,000/population during the years of surveillance

^a^The pre-period was from January 2007 to October 2010

^b^The post-period was from January 2011 to December 2019 and devised in the early post-vaccine period (2011–2014) and the mature post-period (2015–2019)

^c^Other sterile sites (articular fluid, pus/tissues)

### Serotype distribution of pneumococcal isolates

The serotype/serogroup was determined for 95.6% (n = 239) of all invasive isolates recovered from adult and elderly patients (Tables 2 and 3), the remaining strains (11 strains) not serotyped were lost because of freezing problems. In adult population, the most frequent serotypes before vaccination were 19A (n = 9), 8 (n =  7) and 18C (n =  6) whereas after the introduction of vaccination, only serotypes 6B (n =  5) and 3 (n =  5) dominated in the early post-vaccine period and the serotype 3 and 19A with 8.62% (n = 5) each in the mature post period.

**Table 2:** Serotype distribution during the pre- and post-vaccination period (early and mature post-vaccine period) in the adult patients (15–59 years)

Serotype	Pre-vaccine period (2007–2010): N = 71			Early post-vaccine period (2011–2014): N = 57			Mature post-vaccine period (2015–2019): N = 58		
	*n*	%	Cumulative %	n	%	Cumulative %	*n*	%	Cumulative %
4	0	0	0	1	1.75	1.75	0	0	0
6B	1	1.41	1.41	5	8.77	10.52	1	1.72	1.72
9V	1	1.41	2.82	1	1.75	12.28	1	1.72	3.45
14	1	1.41	4.23	1	1.75	14.03	0	0	3.45
18C	6	8.45	12.68	0	0	14.03	0	0	3.45
19F	2	2.82	15.49	1	1.75	15.79	0	0	3.45
23F	3	4.23	19.72	4	7.02	22.8	0	0	3.45
1	3	4.23	23.94	4	7.02	29.82	1	1.72	5.17
5	1	1.41	25.35	0	0	29.82	1	1.72	6.9
7F	2	2.82	28.17	3	5.26	35.08	0	0	6.9
Total PCV-10	20	28.17		20	35.09	*P* = 0.4031	4	6.9	*P* = 0.0021
3	2	2.82	30.99	5	8.77	43.86	5	8.62	15.52
6A	2	2.82	33.8	0	0	43.86	1	1.72	17.24
19A	9	12.68	46.48	0	0	43.86	5	8.62	25.86
Total PCV-13	33	46.48		25	43.86	*P* = 0.7682	15	25.86	*P* = 0.0164
2	1	1.41	47.89	0	0	43.86	0	0	25.86
7A	1	1.41	49.3	2	3.51	47.36	0	0	25.86
7C	1	1.41	50.7	0	0	47.36	0	0	25.86
8	7	9.86	60.56	4	7.02	54.38	4	6.9	32.76
10	2	2.82	63.38	1	1.75	56.14	0	0	32.76
11	0	0	63.38	2	3.51	59.64	1	1.72	34.48
12F	3	4.23	67.61	2	3.51	63.15	0	0	34.48
17F	0	0	67.61	2	3.51	66.66	3	5.17	39.66
22F	0	0	67.61	1	1.75	68.42	1	1.72	41.38
23A	2	2.82	70.42	0	0	68.42	1	1.72	43*.*1
33	0	0	70.42	1	1.75	70.17	3	5*.*17	48*.*27
34	3	4.23	74.65	1	1.75	71.93	1	1*.*72	49*.*99
9N	0	0	74.65	0	0	71.93	1	1*.*72	51*.*71
35F	0	0	74.65	1	1.75	73.68	0	0	51*.*71
NVT	18	25.35	100	15	26.32	100	28	48*.*28	100
Total non PCV-13	38	54		32	56.14	*P* = 0.8096	43	74.14	*P* = 0.0189

**Table 3:** Serotype distribution during the pre- and post-vaccine period (early and mature post-vaccine period) in the elderly patients (≥ 60 years)

Serotype	Pre-vaccine period (2007–2010) N = 15			Early post-vaccine period (2011–2014) N = 26			Mature post-vaccine period (2015–2019) N = 12		
	N	%	Cumulative %	N	%	Cumulative %	N	%	Cumulative %
4	2	13.33	13.33	0	0	0	0	0	0
6B	0	0	13.33	1	3.85	3.85	1	8.33	8.33
9V	0	0	13.33	0	0	3.85	0	0	8.33
14	0	0	13.33	1	3.85	7.69	1	8.33	16.67
18C	0	0	13.33	0	0	7.69	0	0	16.67
19F	0	0	13.33	3	11.54	19.23	0	0	16.67
23F	1	6.67	20	0	0	19.23	0	0	16.67
1	0	0	20	2	7.69	26.92	0	0	16.67
5	2	13.33	33.33	0	0	26.92	0	0	16.67
7F	0	0	33.33	0	0	26.92	0	0	16.67
Total PCV-10	5	33.33		7	26.92	*P* = 0.6692	2	16.67	*P* = 0.33
3	4	26.67	60	6	23.08	50	0	0	16.67
6A	0	0	60	0	0	50	0	0	16.67
19A	0	0	60	1	3.85	53.85	0	0	16.67
Total PCV-13	9	60		14	53.85	*P* = 0.7058	2	16.67	*P* = 0.0255
8	1	6.67	66.66	4	15.38	69.23	1	8.33	25
9N	1	6.67	73.33	0	0	69.23	1	8.33	33.33
12F	1	6.67	80	1	3.85	73.08	0	0	33.33
17F	0	0	80	1	3.85	76.92	0	0	33.33
20	1	6.67	86.66	0	0	76.92	0	0	33.33
23A	0	0	86.66	0	0	0	1	8.33	41.67
22F	1	6.67	93.33	0	0	76.92	0	0	41.67
NVT	1	6.67	100	6	23.08	100	7	58.33	100
Total no PCV-13	6	40		12	46.15	*P* = 0.7058	10	83.33	*P* = 0.026

In adult population, the vaccination coverage of the serotypes included in PCV-10 and PCV-13 showed a non-significant increase from 28.17 to 35.09% (*P* = 0.4) for PCV-10 serotypes and a non-significant slight reduction from 46.48 to 44% (*P* = 0.76) for PCV-13 serotypes in the early post-vaccine period. In the mature post-vaccine period, it significantly decreased to 6.90% (*P* = 0.0021) for the PCV-10 serotypes and to 25.86% (*P* = 0.0164) for the PCV-13 serotypes compared to the pre-vaccine period. In parallel, the rate of NVS did not change in the early post-vaccine period while it increased considerably from 54 to 74.14% (*P* = 0.0189) during the mature post period of vaccination (Table 2). The coverage rate of PCV-13/non PCV-10 serotypes (3,6A,19A) was of 8.77% and 18.96% in the early and mature post-vaccine period respectively. Compared to the pre-vaccine period, there was not a significant difference.

With regard to the elderly population group, there was non-significant difference between the pre-vaccine period and the early post-vaccine period. Compared to the pre-vaccine period, we observed a significant decrease of vaccine coverage of the serotypes included in PCV-13 from 60 to 16.67% (*P* = 0.0255)*.* At the same time, the rate of NVS increased considerably from 40 to 83.33% (*P* = 0.026) during the mature post period of vaccination (Table 3). The coverage rate of PCV-13/non PCV-10 serotypes (3,6A,19A) was 26.93% and 0% in the early and mature post-vaccine period respectively in the elderly population. Compared to the pre-vaccine period, there was not a significant difference.

### Antimicrobial resistance

The rates of resistant isolates to different antibiotics are summarized in Table 4. The comparative analysis of resistance rates to different antibiotics before and after the introduction of vaccination showed a great variability according to age groups. Compared to the pre-vaccine period, there were non-significant changes in antimicrobial resistance rates in the early post-vaccine period for all antibiotics tested in the two age categories (*P *> 0.05), with exception of the rate of cotrimoxazole and penicillin non susceptible pneumococcal isolates. The rate of PNSP decreased significantly from 23.94 to 8.77% (*P* = 0.02) in adult patients in the early post-vaccine period while the rate of cotrimoxazole non susceptible pneumococcal isolates significantly decreased from 29.58 to 8.77% in the early post-vaccine period (*P* = 0.003) and to 7.24% in the mature post-vaccine period (*P* = 0.0007). However, only one case of a strain with reduced sensitivity to ceftriaxone recovered from the adult population, all other strains isolated in the post-vaccination period were sensitive to C3Gs.

**Table 4:** Distribution of Penicillin G-, Erythromycin-, Cotrimoxazole-, Tetracycline-, Chloramphenicol- Ceftriaxone– non susceptible (I + R) strains among adult and elderly patients during pre- and post-vaccination periods

Antibiotic	Adult (15–59 years old)					Elderly (≥ 60 years old)				
	Pre-vaccine period 2007–2010^a^	Post-vaccine period (2011–2019)		p-value		Pre-vaccine period	Post-vaccine period (2011–2019)^b^		p-value	
	2007–2010 (N = 71)	Early post period:2011–2014 (N = 57)	Mature post period: 2015–2019 (N = 69)	(2011–2014)	(2015–2019)	2007–2010 (N = 15)	Early post-vaccine period: 2011–2014 (N = 26)	Mature post-vaccine period: 2015–2019 (N = 12)	(2011–2014)	(2015–2019)
Penicillin G *n* (%)	17 (23.94)	5 (8.77)	11 (15.94)	0.02	**0.23**	2 (13.33)	2 (7.69)	2 (16.66)	0.61	0.81
Erythromycin *n* (%)	5 (7.04)	6 (10.52)	11 (15.94)	0.48	**0.09**	1 (6.66)	2 (7.69)	1 (8.33)	1	0.87
Cotrimoxazole *n* (%)	21 (29.58)	5 (8.77)	5 (7.24)	0.003	**0.0007**	2 (13.33)	2 (7.69)	0 (0.00)	0.61	0.19
Tetracycline *n* (%)	22 (30.98)	11 (19.30)	23 (33.33)	0.13	**0.76**	4 (26.27)	6 (23.08)	3 (25.00)	0.9	0.94
Chloramphenicol *n* (%)	3 (4.22)	6 (10.53)	3 (4.34)	0.29	**0.96**	3 (20.00)	1 (3.85)	1 (8.33)	0.13	0.4
Ceftriaxone *n* (%)	1 (1.41)	1 (1.75)	2 (2.89)	1	**0.54**	0 (0.00)	0 (0.00)	0 (0.00)	NS	NS

I  =  Intermediate, R  =  Resistant

*n*  =  number of (I + R)

NS  =  non-significant

The percentage was calculated as  =  Number of (I + R) × 100/ total number of strains

^a^The pre-period was from January 2007 to October 2010

^b^The post-period was from January 2011 to December 2019 and devised in the early post-vaccine period (2011–2014) and mature post-period (2015–2019)

## Discussion

IPD remain a global public health problem in both children and adults [1, 2]. Vaccination with PCVs recommended by the WHO since 2007 remains the best option to prevent IPD. Nowadays, the vaccination coverage throughout all the country is 100% since 2017 [17]. However, WHO recommends and encourages all the countries that have introduced pneumococcal vaccination in their NIPs to set up an IPD surveillance system to assess the impact of the vaccines. In response, the microbiology laboratory of the IR-UHC has carried out laboratory based surveillance of IPD for more than 25 years [7–9].

Based on obtained results, although the overall incidence rate of IPD in adult patients was < 1/100,000 populations, a significant reduction from 0.71 to 0.54/100,000 populations (*P*  =  0.02) was observed during the early post-vaccine period and decreased to 0.47/100,000 populations in mature post-vaccine period (*P*  =  0.0137) compared to the pre-vaccine period. The use of PCVs in vaccination programmes has also led to IPD incidence reduction among the unvaccinated population due to the herd effect in other countries. These results demonstrate the herd effect following the implementation of vaccination in the NIP in Morocco. However, given there are ~ 100 pneumococcal serotypes and PCVs only include a small number of these, subsequent serotype replacement by NVS may consequently start to cancel out any reduction (possibly substantial) in the overall disease burden initially achieved with the vaccine through both direct and indirect means. As it was the case in our study where the significance decrease in VS was accompanied by a dramatic increase in NVS in the mature post-vaccine period, as reported in many countries where they witnessed the emergence and increase of NVS during the PCV era [18, 19]. It is well established now that paediatric PCVs induce valuable herd protection that extends across the age range but also drive serotype replacement, and these have opposing effects on the overall disease burden [20].

Among the groups most affected by IPD, there is also the elderly population group. Our results showed a non-significant increase in the incidence of IPD during the early post-vaccine period. Contrariwise, there was a non-significance decrease of the incidence rate in the mature post-vaccine period compared to the pre-vaccine period (*P*  =  0.11). This variation may be explained by a natural fluctuation of IPD incidence as already reported by Elmdagrhi et *al.* before vaccination in Casablanca [8]. Whilst Sweden reported no overall decrease in total IPD in those aged  >  65 years old, vaccine type IPD in this age group did decrease [21]. Ireland similarly saw a decrease in cases of PCV7 vaccine serotype IPD in adults aged  >  65 years in the post introduction of vaccine in the paediatric programme [22].

However, the absence of a herd effect in the older population has been reported in several countries [21–23]. In contrast, in Australia, a reduction of 71% (95% CI: 36%; 88%) was observed in the post-vaccination period in people over 60 years of age, reflecting the herd effect [24]. As the herd effect increases with the time since the introduction of vaccination in the NIP, we expect even greater indirect protection of that age group in the coming years. Furthermore, these data suggest an alternative solution to this age group. New higher valent PCVs could be considered for protecting Moroccan adults against pneumococcal diseases. Nowadays, adult PCV15 and PCV20 are now licensed (EU/US). Besides PCVs, The Pneumococcal Polysaccharide Vaccine (PPV), PPV-23, has been recommended in several countries around the world, but the Moroccan NIP does not include adult pneumococcal vaccination. Djennad et *al.* (2019) [25] supports limited/moderate short-term effectiveness of PPV23 against vaccine type IPD in those aged  >  65 years which aligns with the consensus that PPV23 provides some protection against IPD in older adults, as is stated by Wang et al. [26]. However, evidence that PPV23 is effective against community acquired pneumococcal pneumonia in older adults continues to remain very inconsistent.

Furthermore, in some countries, especially in Europe, certain serotypes that are common in one age group, but rare in others, are increasing in incidence in groups where they were rare. This is the case in Finland with serotype 11A common in adults increasing in children, in France with serotypes 8 and 9 N common in adults increasing in children, serotypes 10A and 23B common in children increasing in adults and in Norway with serotype 24F common in children increasing in adults; serotypes 8 and 9 N commonly found in adults increasing in children [27]. In our study in Casablanca, Morocco, no serotype was particularly dominant in any age group.

In addition to the impact on IPD and serotype distribution, PCVs have been shown to be effective in reducing the prevalence of resistant strains in the countries where they have been introduced. In the present study, in contrast to the elderly population group, the prevalence of PNSP strains decreased from 23.94 to 8.77% during the early post-vaccine period (*P*  =  0.02) and to 15.94% in mature post-vaccine period (*P*  =  0.23) compared to the pre-vaccine period in adult population, while the strains with decreased susceptibility to co-trimoxazole decreased significantly both from 29.58 to 8.77% in early post-vaccine period and to 7.24% in mature post-vaccine period. As reported by Diawara et al., the rate of co-trimoxazole-resistant strains decreased following vaccination even in the children population [9]. Variable rates of PNSP strains have been reported in several regions. In Malaysia, the rate of PNSP strains was 22.4% between 2014 and 2017 [28], 27.5% in Iran between 2017 and 2019 and 75.3% in Tunisia between 2012 and 2016 [29]. Overall, the highest rates are reported in Africa (64.3%), the Middle East (46.4%) and North America (38.5%) [30].

It is important to note that this rate does not vary only by region and period of surveillance, it also depends on circulating serotypes. In Japan, antimicrobial susceptibility testing revealed that 88.9% and 89.4% of serotype 35B and 15A strains respectively were of PNSP [31]. In Tunisia, the two most frequent serotypes 19F and 14 represented higher rates of PNSP strains with 18% and 29.4% respectively [29]. In Casablanca, vaccination has significantly reduced the prevalence of multi-antibiotic resistant serotypes; PNSP strains were often associated with some pre-vaccine era serotypes covered by PCV including serotypes 9 V, 6B, 14, 19A, 19F and 23F [9]. As a result, the low rate observed in Casablanca during our study period would be linked to the persistence of 6B serotype and 19A in the early and mature post-vaccine period respectively. The study conducted in Taiwan showed that although the overall rate of β-lactam non-susceptible *S. pneumoniae* strains decreased in 2010 due to the decline of serotypes 19F and 23F, it has increased since 2012, partly due to the rise of serotypes 15A and 23A [32]. The rate of ceftriaxone non-susceptible *S. pneumoniae* strains remains very low but very concerning. Given that ceftriaxone remains a reference choice for the empirical treatment of IPD, selective pressure through overuse of this antibiotic could lead to increased rates of PNSP strains to this molecule as reported elsewhere [33]. However, regional study, maybe a national multisite scale study would be important for the future to highlight the country’s situation.

### Limit of the study

Our study is a one site laboratory-based surveillance of invasive pneumococcal isolates. Regional study or a national multisite scale study would be important to highlight the serotype distribution and the antimicrobial susceptibility of invasive *Streptococcus pneumoniae* isolates in the whole country.

## Conclusions

Although childhood vaccination has considerably reduced the incidence of IPD in adults aged 15–59 years through the herd effect, IPD remain a real public health problem following the alarming increase in NVS. The rate of antibiotic resistance was relatively low. Nevertheless, resistance constitutes a serious problem to the therapeutic arsenal due to the known capacity for genetic dissemination in the pneumococcus. Continued surveillance should continue to assess the changes in serotype distribution of invasive and non-invasive *S. pneumoniae* in the mature post-vaccine period.

## Data Availability

All the data supporting the conclusions of the present study are included within the manuscript. Supplementary datasets used and/or analyzed during the current study are available from the corresponding author on reasonable request.

## Abbreviations

ARR: Absolute risks reduction
C3G: Third-generation cephalosporins
CDC: Disease Control and Prevention
CI: Confidence interval
CLSI: Clinical and Laboratory Standards Institute
CSF: Cerebrospinal fluid
EUCAST: European Committee on Antimicrobial Susceptibility Testing
I: Intermediate
IPD: Invasive pneumococcal diseases
IR-UHC: Ibn Rochd University Hospital Centre
MIC: Minimum inhibitory concentration
NIP: National immunization program
NVS: Non-vaccine serotypes
PCV: Pneumococcal conjugate vaccine
PNSP: Penicillin-non-susceptible pneumococci
R: Resistant
RRR: Relative risks reduction
*Streptococcus pneumoniae*: *S. pneumoniae*
VS: Vaccine serotypes
